# miR-26a induced the suppression of tumor growth of cholangiocarcinoma via KRT19 approach

**DOI:** 10.18632/oncotarget.13229

**Published:** 2016-11-09

**Authors:** Ping Wang, Long Lv

**Affiliations:** ^1^ Department of General Surgery, People's Hospital of Gaochun, Gaochun, Nanjing, 211300, Jiangsu Province, China; ^2^ Department of Endocrinology, People's Hospital of Gaochun, Gaochun, Nanjing, 211300, Jiangsu Province, China

**Keywords:** proliferation, cholangiocarcinoma, stem cell, miRNA, KRT19

## Abstract

**Background and Aims:**

KRT19 was identified as one of the key biomarkers for distinguishing cholangiocarcinoma (CCA) and hepatocellular carcinoma. The detailed role of miRNAs involved in the oncogenic incident of KRT19 was poor investigated.

**Results:**

Based on prediction and validation, miR-26a was inversely correlated with KRT19 in patients’ tissues samples and biopsies. Ectopic expression of miR-26a dramatically suppressed cell proliferation and tumor growth *in vitro* and *in vivo*. Knock-down miR-26a could induce an increasing population of SP cells by promoting KRT19 expression. The KRT19 was also suppressed via directly binding at 3′UTR region by miR-26a.

**Materials and Methods:**

Bioinformatics prediction was first applied to screening the potential miRNA involved. RT-PCR, Immunohistochemistry and Western blot were used to examine the expression of miRNAs and candidate genes in 65 pairs of cholangiocarcinoma. The loss-and gain-function assay was employed to detect the role of certain miRNA *in vitro* and *vivo*. Side-population (SP) cells were detected and sorted by flow cytometry.

**Conclusions:**

Aberrant decreased miR-26a could promote cell proliferation by regulating KRT19 which play important roles in the pathogenesis of CCA.

## INTRODUCTION

Cholangiocarcinoma (CCA), as a biliary tract cancer originating in the epithelium of the biliary tree, was the second most common primary liver malignant tumor after hepatocellular carcinoma (HCC) and was generally divided into three subgroups including intrahepatic cholangiocarcinoma (ICC), hilar cholangiocarcinoma and extrahepatic cholangiocarcinoma (ECC) [1–3]. According to public data, the incidence and mortality of CCA was rising over the past decade especially in China [4, 5]. However, the detailed mechanisms of the pathogenesis of CCA still remain poor.

KRT19 was revealed as marker for cholangiocytes, hepatic progenitor cells (HPCs) and early hepatoblasts. It has been identified that the existence of KRT19 was linked with a poor prognosis for patients diagnosed with CCA [6–8]. Researchers has found that KRT19 was involving in tumor cell proliferation and invasion promotion not only in HCC, but also in CCA in a Caucasian series of 242 consecutive HCC samples in comparison with other biliary/HPC markers, epithelial cell adhesion molecule (EpCAM) and α-fetoprotein (AFP) revealed [9–11].

MiRNAs were a set of small, noncoding RNAs with 19-25nt that inversely regulated the gene expression by binding to specific target sites in the 3′ Untranslated Regions (UTR) of target mRNA [12, 13]. MiRNAs could act as either oncogene or tumor suppressor gene by regulating cell proliferation, migration, apoptosis and differentiation through a post-transcription level regulation [14, 15]. Reportedly, several studies have identified some dysregulated miRNAs in CCA patients [16, 17]. In addition, miRNAs were also participating in the development of CCA such as lymph node metastasis [18]. Serum circulating miRNAs were promising fingerprints for CCA for which the best chance of successful treatment is timely diagnosis and management [19].

In this study, we searched the database including miRbase, Target Scan, PicTar and miRNA Target to found the candidate miRNAs which might associate with KRT19. The further loss-and gain-function approaches was also applied *in vitro* and *in vivo*.

## RESULTS

### Decreased miR-26a inversely associated with KRT19 in CCA patients

**Figure 1:**
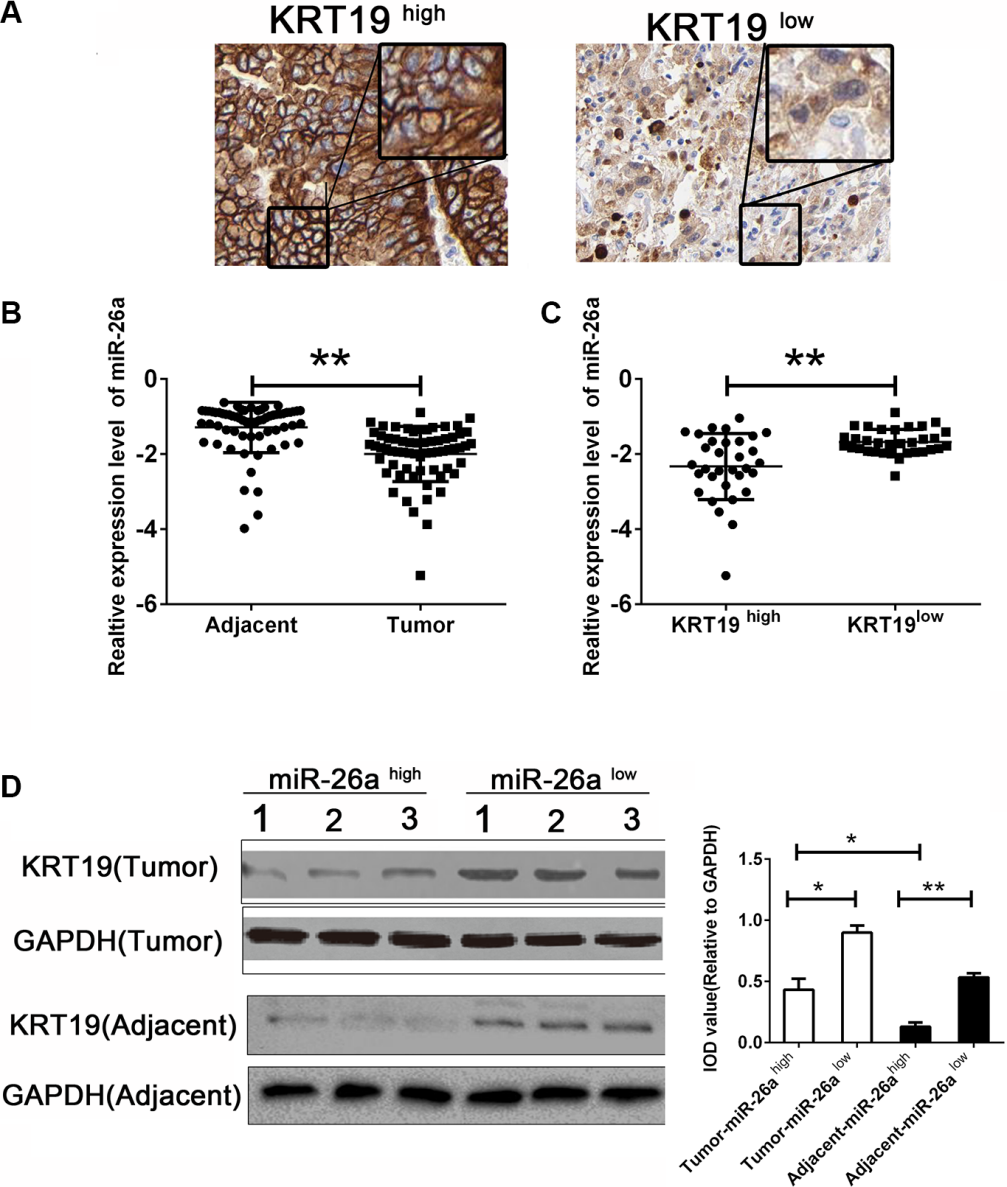
miR-26a inversely correlation with KRT19 (**A**) Representative stain of KRT19 in tumor tissues biospy of CCA patients. (**B**) Relative expression levels of miR-26a in human CCA tumor tissues and adjacent corresponding tissues (*n* = 65) were evaluated by qRT-PCR. (**C**) Relative expression of miR-26a in CCA patients of KRT19^high^ and KRT19^low^ subgroup. (**D**) Western blot of KRT19 in CCA patients of miR-26a^high^ and miR-26a^low^ subgroup. Data were presented as Mean and range of log-transformed relative expression level. **indicates significant difference (*P <* 0.01).

We first detected the expression of KRT19 in human tissue biopsies obtained from CCA patients by immunohistochemistry. As presented in Figure 1A, we found an aberrant distribution of KRT19 in CCA patients. We next predicted the potential miRNAs which might be associated with KRT19 expression and bound with KRT19 in four independent databases. As presented in Table 1, six miRNAs was considered as candidate. Further RT-PCR was conducted in 65 paired RNA samples from CCA patients to examine the abnormal expression of candidate miRNAs. Among the six candidates, we found that miR-26a was remarkable decreased in the tumors tissues of CCA patients comparing with the corresponding adjacent (Figure 1B); the rest remains no difference between the two groups (data not shown). To further document the correlation between miR-26a and KRT19, we divided the CCA patients into two subgroups (KRT19^high^ and KRT19^low^) by using the medium of KRT19 expression in tumor tissues as cutoff. Interestingly, miR-26a was deeply decreased in patients with a higher expression of KRT19 (Figure 1C). The protein level detection also confirmed the inversely correlation in CCA patients in both tumor tissues and corresponding adjacent tumor tissues (Figure 1D). Based on the clinical information analysis, we found that the miR-26a and KRT19 was highly associated with the tumor size of patients, indicating that miR-26a might act an important role in the tumorigenesis of CCA (Table 2).

**Table 1:** Bioinformatics prediction for potential candidate miRNAs for KRT19

Target gene	Related Gene	miRbase	Target Scan	PicTar	miRNA Target
**KRT19**	miR-26a	√	√	√	√
	miR-298	√	√		√
	miR-922		√	√	√
	miR-1912		√	√	√
	miR-4482	√	√	√	
	miR-8080	√	√	√	

**Table 2:** The clinicopathological relevance analysis of miR-26a and KRT19 expression in cholangiocarcinoma patients

	miR-26a			KRT19		
Characteristic	Low	High	*P*value	Low	High	*P*value
**All cases**	33	32		32	33	
**Age**			0.714			0.714
<60	15	16		16	15	
≥60	18	16		16	18	
**Gender**			0.725			0.228
Male	22	20		23	19	
Female	11	12		9	14	
**Differentiation grade**			0.897			0.367
Well	21	22		23	20	
Moderate	8	7		5	10	
Poorly	4	3		4	3	
**Tumor Size (cm)**			0.035			0.002
≤5 cm	12	20		22	10	
>5 cm	21	12		10	23	
**Tumor Origination**			0.915			0.995
Left	13	11		12	12	
Right	19	20		19	20	
Bilateral	1	1		1	1	
**TNM stage**			0.694			0.371
I–II	18	19		20	17	
III	15	13		12	16	
**Metastasis**			0.909			0.379
Yes	18	17		19	16	
No	15	15		13	17	

### Function assay of miR-26a *in vitro* and *in vivo*

**Figure 2:**
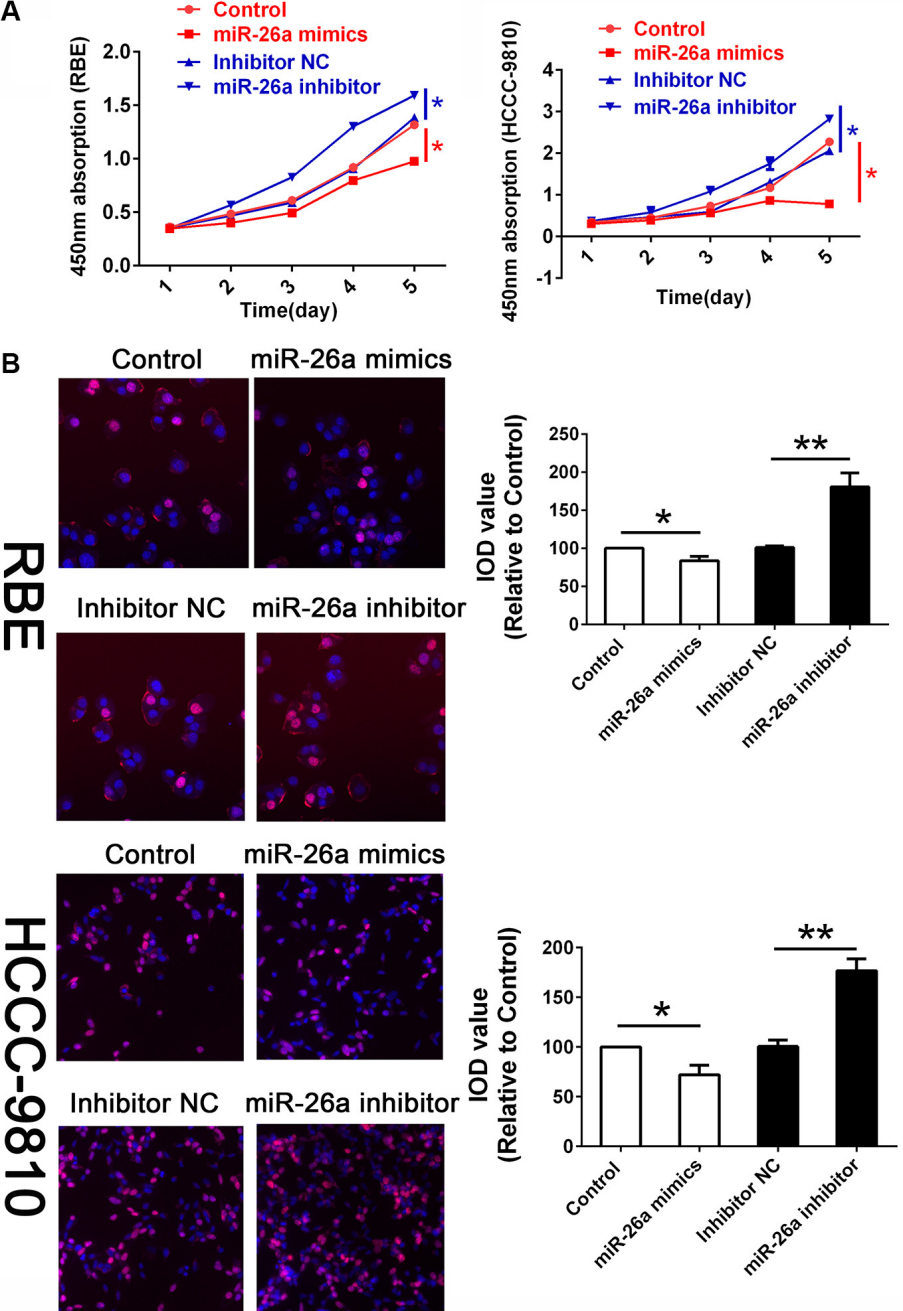
miR-26a suppressed cell proliferation *in vitro* (**A**) CCK8 assay were employed to detecting the proliferation of CCA cells. Data was presented with Mean ± SE. *indicates significant difference compared with that of control cells (*P <* 0.05). (**B**) The EDU stain also performed in cells treated as described above with a magnification of 200. The integral optical density value of cells treated with control plasmids was normalized to 100%.

According to the data collected above, we then examined the function of miR-26a in both RBE and HCCC-9810 cell lines. The expression of miR-26a as well as KRT19 was confirmed firstly (Supplemenary Figure S1). The CCK8 assay revealed that the absorption in 450 nm of cells was statistically suppressed by treating cells with miR-26a mimics while could be attenuated by treating cells with miR-26a inhibitor when monitored the proliferation for five days (Figure 2A). In addition, the proliferative cells were also measured by EDU assay treating with different groups after 48 hours. As presented in Figure 2B, we found that when normalized with DAPI, the percentage of cells in the logarithmic phase was reduced when ectopic expressing miR-26a in both RBE and HCCC-9810. The proliferative cells could also been elevated by suppression the expression of miR-26a (Figure 2B). Since miR-26a has been mentioned associating with cell invasion in human cancers [20], we also measured cell invasion by using transwell assay. As presented in Supplementary Figure S2, no significant difference was obtained among each group.

**Figure 3:**
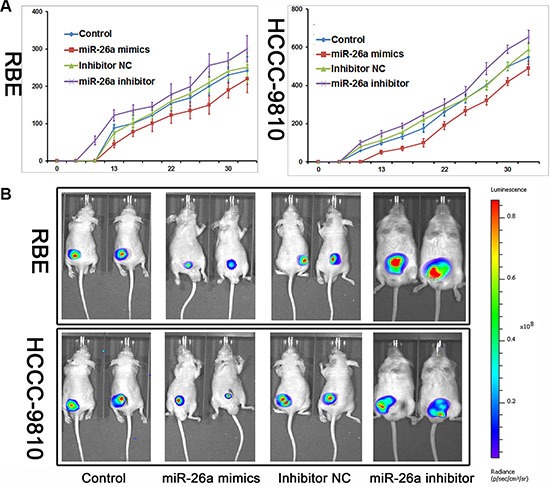
miR-26a decreased tumor growth *in vivo* (**A**) Nude mice were subcutaneously transplanted with cells stably expressed with miR-26a mimics/Inhibitor or control (*n* = 5). The volume of each tumor was calculated as the length × width^2^ × 0.5. The unit of y-coordinate was mm^3^. (**B**) Mice with established tumors in different groups were imaged every 7 days with the IVIS Lumina II system; the images shown were taken on day 25.

The *in vivo* tumor growth activity assay on CCA was further examined in the xenograft model in nude mice. Among the cell lines tested, both the subcutaneous inoculation of RBE and HCCC-9810 cells into nude mice caused the tumor burden at the site of injection in all mice. The sizes of tumors formed in mice treated with miR-26a mimics were significantly smaller than those of the control. Consistently, cells treated with miR-26a inhibitor caused a promoted tumor growth comparing with the inhibitor control group (Figure 3A, 3B).

### MiR-26a targeting side population cells in CCA

**Figure 4:**
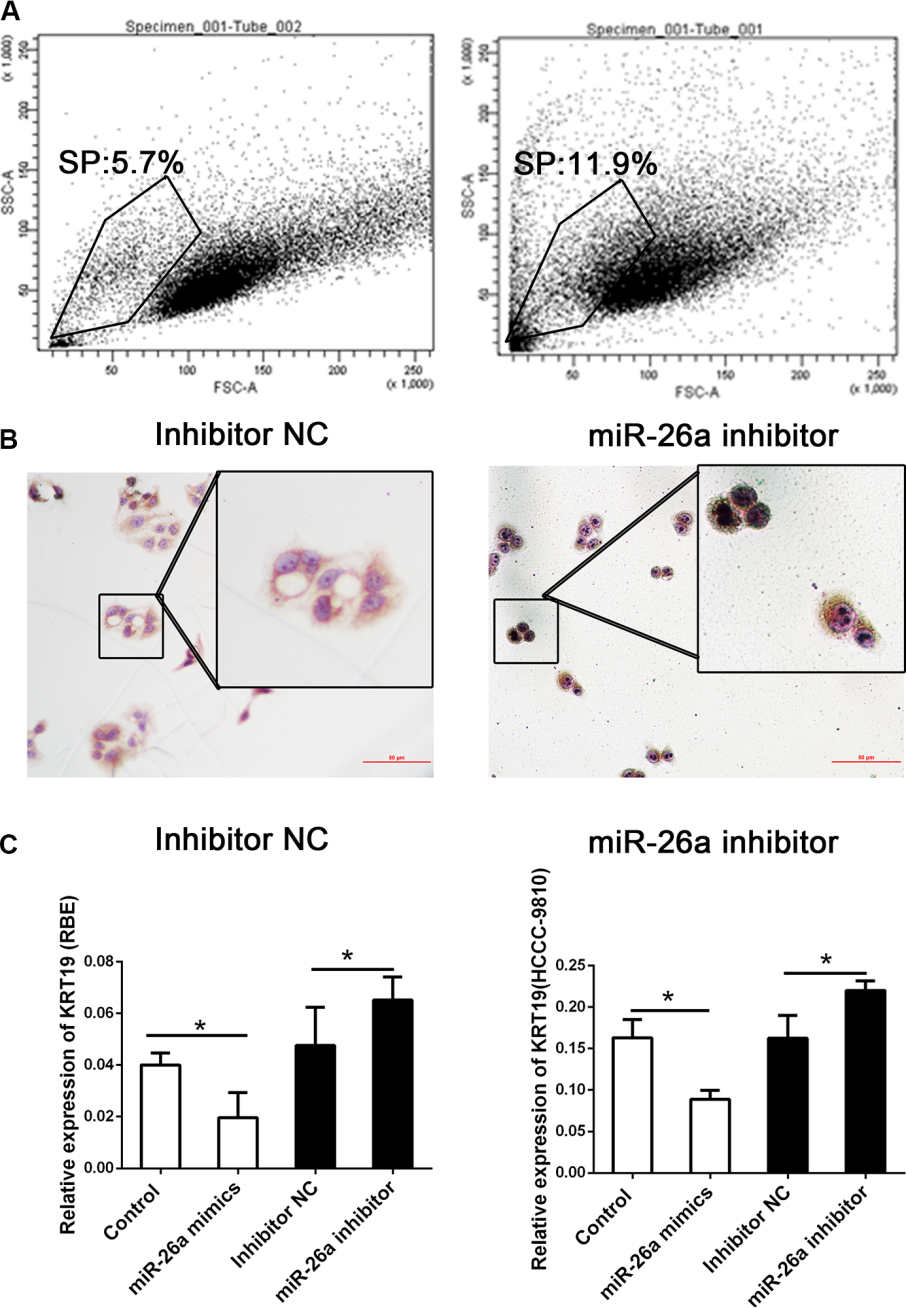
Decreased miR-26a could promote the distribution of side-population cells (**A**) The percentage in the SP cells detected by flow cytometry (**B**) Immunocytochemistry on cytosine of sorted cells targeting KRT19. Scale bar: 200 mm; magnification scale bars 100 mm. (**C**) Relative expression of KRT19 in cells treated with miR-26a mimics, inhibitor or controls. *indicates significant difference compared with that of control cells (*P <* 0.05). All tests were performed in triplicate and presented as mean ± SE.

Previous studies have indicated that KRT19 contributed toward the maintenance of cancer stem cells (CSCs) [21]. The side population (SP) cells are a rare subset of cells enriched with CSCs and has also been used to exploring the stem-like function of KRT19 [22]. The SP phenotype has been proven to be invaluable for stem cell isolation in the absence of definitive cell surface markers [23, 24]. We considered that if miR-26a was associated with KRT19, the SP distribution might be also altered by the dysregulation of miR-26a. We next examined the proportion of SP cells in RBE cells with different treating. ABC transporter activity was inhibited using verapamil hydrochloride as the control group. In samples without any treating, about 5.7% of SP cells were stained positively. In the cells treating with miR-26a inhibitor, more than 10% of SP cells were detected (Figure 4A). We also tried to detect the SP population in cells overexpressed with miR-26a; however, the percentage of SP cells was difficult to distinguish from the verapamil normalization as well as further sorting.

Next, we sorted the SP cells and conducted the ICC assay targeting KRT19 to further confirm the different distribution of SP. We found that alone with the decreased number of SP cells, the KRT19 was also increased in those cells (Figure 4B).

### KRT19 was the direct targets of miR-26a

**Figure 5:**
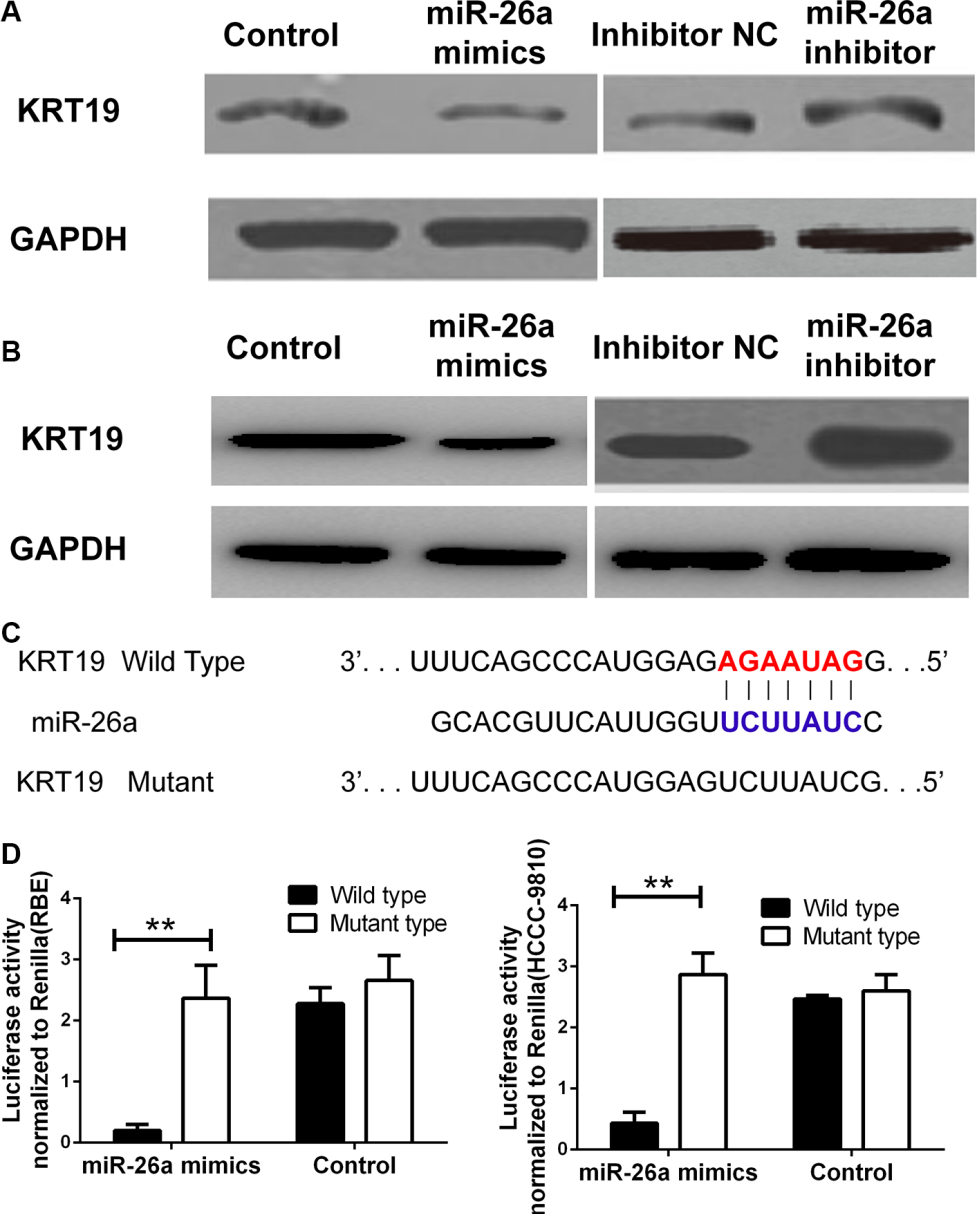
miR-26a could directly binding with KRT19 3′UTR The protein expression levels of KRT19 were detected by western blot in cells treated with miR-26a mimics, inhibitor or controls (**A**) RBE cell line, (**B**) HCCC-9810 cell line. (**C**) The sequence alignment of human miR-26a with 3′ UTR of KRT19. Bottom: mutations in the 3′-UTR of KRT19 in order to create the mutant luciferase reporter construct. (**D**) Cells were co-transfected with miR-26a mimics or Control, Renilla luciferase vector pGL3-SV40 and KRT19 3′UTR luciferase reporters for 48 h. Both firefly and Renilla luciferase activities were measured in the same sample. Firefly luciferase signals were normalized with Renilla luciferase signals. **indicated remarkable significant difference (*P <* 0.01). All tests were performed in triplicate and presented as mean ± SE.

Since miR-26a could suppress the cell proliferation and SP population in CCA cell lines, the detailed mechanism still remains unclear. Based on the regulation pattern of miRNAs in human carcinoma reported, we detected the mRNA and protein expression of KRT19 in the two cell lines treating with miR-26a mimics or inhibitor. As present in Figure 4C, the mRNA expression of KRT19 was decreased in cells treating with miR-26a mimics and could be upregulated by inhibiting miR-26a expression. The regulation was also confirmed by western blot (Figure 5A, 5B).

Furthermore, The miRNA luciferase reporter assay by constructing the wild type and mutant type luciferase reporter plasmids containing the binding region of the 3′UTR of KRT19 mRNA were employed (Figure 5C). We found that co-transfection of miR-26a mimics and pGL3-KRT19 3′UTR wild type reporter plasmids significantly reduced the luciferase activity in cell lines, as compared with the control and the mutant type, suggesting miR-26a directly targets KRT19 (Figure 5D).

## DISCUSSION

To our knowledge, It was the first time for us reporting the function role of miR-26a in cholangiocarcinoma through regulation of KRT19 approach. We have identified miR-26a as an important tumor suppressor of cholangiocarcinoma growth both *in vitro* and *in vivo.* We also found that treating with miR-26a might suppress CCA tumor growth in xenografts, suggesting a therapeutic potential for CCA patients in the future. Most importantly, we provided evidence that the miR-26a might suppress the tumor growth via decreasing the side population cell percentage resulting in the inhibition of stem-like cells in CCA.

Numerous studies suggested that miRNAs played an important role in the pathogenesis of tumor. It was reported that the dysregulation of miRNAs participated in CCA progression acting as either suppressors or promoters at the post-transcriptional regulation stage. For example, lower expression of miR-122 in CCA tissues, and higher expression of miR-122 could inhibit proliferation, stimulate apoptosis and suppress invasion of tumor cells, indicating that miRNA might be a new target for CCA diagnosis or treatment [25]. In addition, miR-21, as a oncogene, promoted cell proliferation and growth *in vivo* and *in vitro* by directly targeting PTPN14 and PTEN mRNAs in CCA [26, 27].

Among the various miRNAs identified in human malignant tumors, miR-26a was confirmed as an important role in the development of cancer. Lu, et al. found that lentiviral overexpression of miR-26a in ZOS and 143B osteosarcoma cells decreased the expression of stem cell markers and suppressed sarcosphere formation, as well as ALDH activity [28]. Furthermore, Sun, et al. also indicated that miR-26a may act on the 3′UTR of CDC6 to regulate CDC6 expression, which then inhibit the proliferation of ovarian cancer cells and induce cell apoptosis [29]. This important found was also confirmed by an independent study [30]. All of this indicated that miR-26a was acting as a tumor suppressor gene. Since miR-26a was identified to inhibit invasion in osteosarcoma cells by targeting PFKFB3 and also target COX-2. Here we found no difference of cell invasion with different treating; this might be because of the low expression abundance of PFKFB3 or COX-2 which lead the incapacitation in affecting cell invasion

As we known, KRT19 was not regarded as an oncogene or tumor suppressor gene in human disease. Specifically, the expression of KRT19 was also reported in nonmalignant cells. In human malignant tumor, KRT19 was associated with the proliferation and invasive behavior of tumor cells. The absence of KRT19 could reduce the growth and metastasis of HCC *in vivo* through a “stemness” function [31, 32]. The KRT19-associated gene signature showed a strong overlap with that of other previously described more malignant HCC subclasses, such as poor survival or proliferation of HCC subtypes.

## CONCLUSIONS

The aberrant expression level of miR-26a in CCA patients was high associated with tumor growth. Based on our founding, miR-26a may associate with the proliferation of CCA cells by down-regulated KRT19. The important role of miR-26a might provide a theoretical basis of a novel targeted therapy for CCA.

## MATERIALS AND METHODS

### Patient and tissue samples

65 paired CCA tissues were obtained after routine surgery. Immediately snap frozen in liquid nitrogen, and stored at −80°C until RNA and protein extraction. Informed consent for tissue analysis was obtained prior to surgery; the study was approved by our Institutional Ethics Committee. All the tissues used in this study was confirmed as CCA through pathological diagnosis. The tumors were classified according to World Health Organization classification. This study was approved by the Ethical Committee of The People's Hospital of Gaochun and every patient had written informed consent.

### Cell culture and transfection

CCA cell lines including RBE and HCCC-9810 were purchased from the Chinese Academy of Sciences Cell Bank (Shanghai, China). All cells were cultured in RPMI-1640 (Gibco, USA) supplemented with 10% fetal bovine serum (FBS) (Invitrogen, Carlsbad, USA) and grown in humidified 5% CO2 at 37°C. MiR-26a mimics, inhibitor and relative controls were obtained from Genepharma (Shanghai, China). The transfection was conducted by using Lipofectamine 2000 (Invitrogen Corp, CA, USA) as descripted previously [32].

### Bioinformatics analysis

The bioinformatics analysis was employed by using the miRNA associated target genes prediction database. We searched the potential miRNAs which might target KRT19 in Targetscan (www.targetscan.org/), PicTar (pictar.mdc-berlin.de/) and miRTarget (mirdb.org/) [33]. The mature sequence of KRT19 3′UTR was used as the template. The candidate target genes were screened according to the rank score of database.

### Quantitative RT-PCR

RNA purity and concentration were detected by UV spectroscopy while RNA integrity was evaluated using agarose gel electrophoresis. The cDNA template was got by reverse transcription (RT) step using NCodeTM miRNA First-Strand complementary DNA synthesis kit, and then Fast SYBR Green Master Mix was used for real-time PCR with ABI 7900 real-time PCR system (ABI, CA, USA) according to the protocol reported [18]. Real-time PCR protocol included an initial denaturation step (95°C for 5 min) and 40 cycles of denaturation (95°C for 10 s), annealing (60°C for 20 s) and extension (72°C for 10 s). The relative expression level of miR-26a was calculated using 2^−ΔΔCt^ method.

### Protein analysis

Total proteins extracted from tissues or cultured cells using RIPA buffer containing PMSF were used for western blot (Roche, Basel, Switzerland). Equal amount of proteins (100 ug) were separated with 7.5%/12.5% sodium dodecyl sulphate polyacrylamide gel electrophoresis (SDS-PAGE) and transferred to polyvinylidene fluoride (PVDF) membrane. The blots were developed using ECL reagent (Millpore, MASS, USA). Equal amount of protein loading in each lane was confirmed and normalized using GAPDH antibody.

### Cell proliferation assays

CCK8 assay was applied to detect cell proliferation. Absorbance at 450 nm was measured by the TECAN infinite M200 Multimode microplate reader (Tecan, Mechelen, Belgium). EDU (5-ethynyl-2′-deoxyuridine) assay was used. The red stain indicted the proliferative cells. Each assay was performed in triplicate and repeated three times independently.

### Dual-luciferase reporter assay

The 3′-UTR sequence of KRT19 predicted to interact with miR-26a or a mutated sequence with the predicted target sites were inserted into pGL3 promoter vector (Genscript, Nanjing, China). A Renilla luciferase vector pRL-SV40 (5 ng) was also co-transfected to normalize the differences in transfection efficiency. Transfection was repeated three times in triplicate.

### Xenograft model in nude mice

Xenograft tumors were generated by subcutaneous injection of RBE and HCCC-9810 cells (2 × 10^6^) ,stablely treating with miR-26a mimics or inhibitor, into the hind limbs of 4–6 week old BALB/C athymic nude mice (nu/nu; *n* = 5 for each group). All mice were housed and maintained under specific pathogen-free conditions. All experiments were approved by the Animal Care and Use Committee and performed in accordance with institutional guidelines. Tumor size was measured using a slide caliper and tumor volume was determined by the formula: 0.5 × A × B^2^ and was monitored by the IVIS@ Lumina II system (A: length, B: width).

### Side-population cell detection and sorting

Cells were incubated with the transport blockers verapamil (100 μM; Sigma-Aldrich, MO, USA) 20 minutes prior to the Hoechst33342 incubation. After that, the Propidium Iodide (2 μg/ml; Sigma-Aldrich, MO, USA) as markers for viable cells was added. The cell suspensions were analyzed using a FACSAriaII (BD Biosciences, CA, USA). The SP was visualized after UV excitation on the basis of blue emission through a 424/44 filter and of red emission through a 630/22 filter (Omega Optical, Brattleboro, VT). Within the living cell population (propidium iodide negative), the side and main population (MP) were sorted separately and collected.

### Statistical analysis

All the experiments were repeated for three times independently. Numerical data were presented as Means ± standard deviation (SD). Experimental data of tissue samples were presented as box plot of the median and range of log-transformed which was analyzed by Wilcoxon rank-sum (Mann-Whitney) test. While the results obtained from experiment *in vitro* assays were analyzed by double-sided Student's *t-test*. Results were considered statistically significant at *P <* 0.05.

## SUPPLEMENTARY MATERIALS



## Abbreviations

CCA: cholangiocarcinoma
UTR: untranslation region
RT-PCR: real time: polymerase chain reaction
SDS-PAGE: sodium dodecyl sulphate polyacrylamide gel electrophoresis
PVDF: polyvinylidene fluoride
EDU: 5-ethynyl-2′-deoxyuridine
SP: side population.
