# Supplementary material for: miR-26a induced the suppression of tumor growth of cholangiocarcinoma via KRT19 approach
Source: Oncotarget. 2016 Nov 9;7(49):81367–76. doi: 10.18632/oncotarget.13229 (PMC5348398; doi:10.18632/oncotarget.13229)
Supplement: Supplementary file 1 [file oncotarget-07-81367-s001.pdf]

miR-26a induced the suppression of tumor growth of cholangiocarcinoma via KRT19 approach

Supplementary Materials

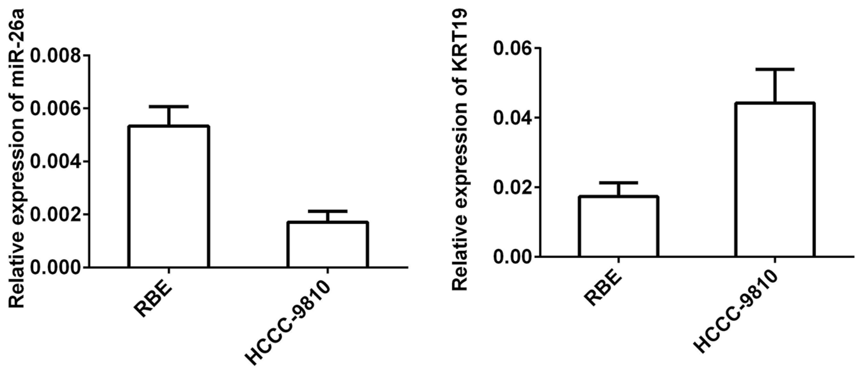

Supplementary Figure S1: Relative expression of miR-26a and KRT19 in human CCA cell lines. (**A**) Relative expression of miR-26a in human CCA cell lines. (**B**) Relative expression of KRT19 in human CCA cell lines. Data were presented as the mean ± SEM.

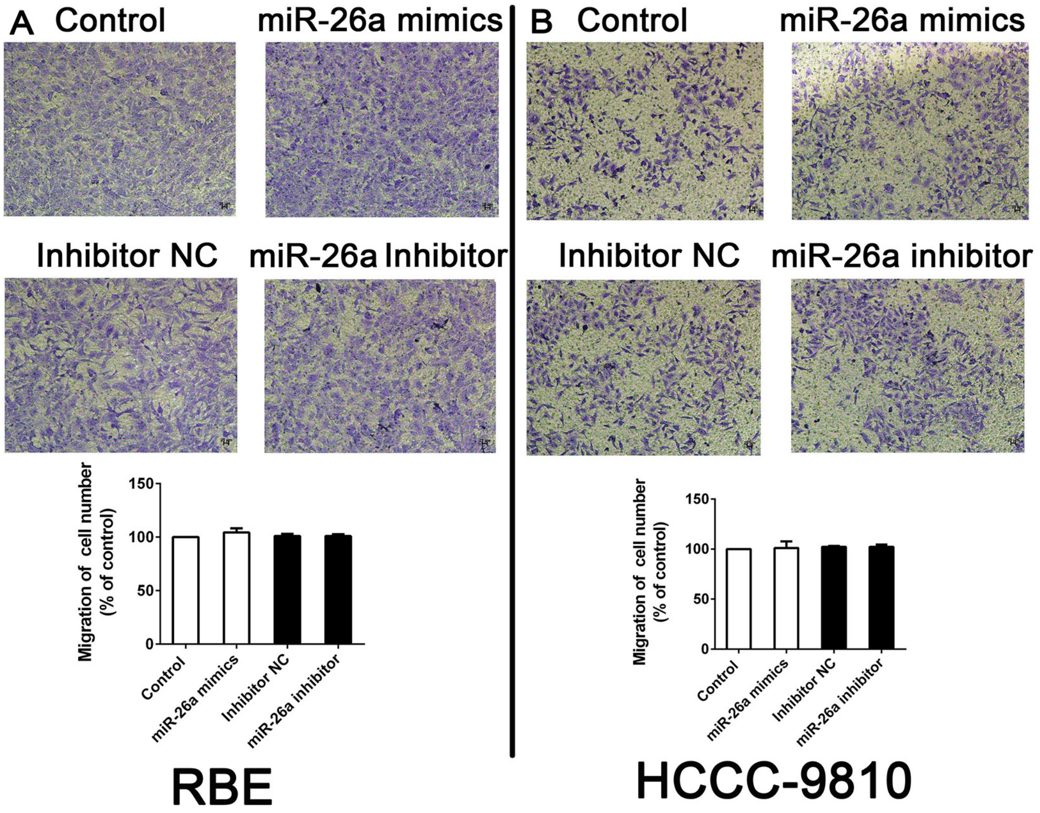

Supplementary Figure S2: No significant of miR-26a on cell invasion. Cells were treated with shRNA of miR-26a mimics, inhibitor and corresponding controls. The Transwell assay with matrigel was conducted to measure the cell invasion. The migrated cells was normalized with control group. (**A**) RBE cell line. (**B**) HCCC-9810 cell line.

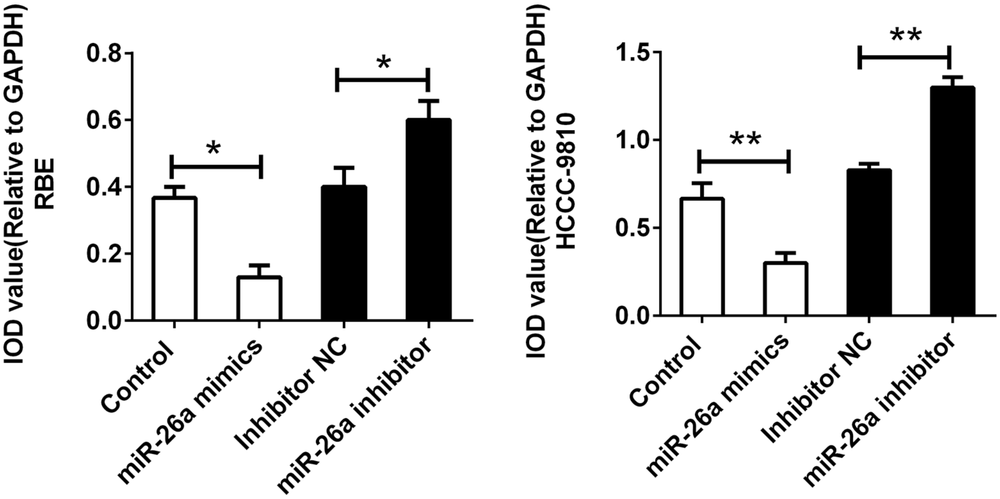

Supplementary Figure S3: The integral optical density analysis of western blot. The integral optical density of band detected by western blot was measure and normalized with GAPDH. Data were presented as the mean ± SEM. *indicates significant difference ($P < 0.05$), **indicates remarkable significant difference ($P < 0.01$).
